# Exploring the potential of PROCOSINE and close-range hyperspectral imaging to study the effects of fungal diseases on leaf physiology

**DOI:** 10.1038/s41598-018-34429-0

**Published:** 2018-10-29

**Authors:** Julien Morel, Sylvain Jay, Jean-Baptiste Féret, Adel Bakache, Ryad Bendoula, Francoise Carreel, Nathalie Gorretta

**Affiliations:** 1 UMR ITAP, Irstea, Montpellier SupAgro, Univ. Montpellier, Montpellier, France; 2 Department of Agricultural Research for Northern Sweden, Swedish University of Agricultural Sciences, Umeå, Sweden; 3 Aix-Marseille Univ., CNRS, Central Marseille, Institut Fresnel, Marseille, F-13013 France; 4 UMR TETIS, Irstea, Univ. Montpellier, Montpellier, France; 5 UMR AGAP, Cirad, Montpellier, France

## Abstract

The detection of plant diseases, including fungi, is a major challenge for reducing yield gaps of crops across the world. We explored the potential of the PROCOSINE radiative transfer model to assess the effect of the fungus *Pseudocercospora fijiensis* on leaf tissues using laboratory-acquired submillimetre-scale hyperspectral images in the visible and near-infrared spectral range. The objectives were (i) to assess the dynamics of leaf biochemical and biophysical parameters estimated using PROCOSINE inversion as a function of the disease stages, and (ii) to discriminate the disease stages by using a Linear Discriminant Analysis model built from the inversion results. The inversion results show that most of the parameter dynamics are consistent with expectations: for example, the chlorophyll content progressively decreased as the disease spreads, and the brown pigments content increased. An overall accuracy of 78.7% was obtained for the discrimination of the six disease stages, with errors mainly occurring between asymptomatic samples and first visible disease stages. PROCOSINE inversion provides relevant ecophysiological information to better understand how *P. fijiensis* affects the leaf at each disease stage. More particularly, the results suggest that monitoring anthocyanins may be critical for the early detection of this disease.

## Introduction

Plant diseases are a major issue for food and crop production, and can lead to yield gaps between potential and actual productions^1^. One way to reduce those gaps is to develop efficient and accurate monitoring systems to detect plant diseases. To date, most of those monitoring systems rely on identification based on visual assessment and expert knowledge, which is subjective, time- and money-consuming. Other methods using molecular analyses techniques such as polymerase chain reaction (PCR) have recently gained importance^2^, but remain time-consuming and highly disease-specific, making them inappropriate for processing numerous plant samples.

Remote sensing has gained tremendous importance in agriculture, providing objective data that can be used to retrieve information on the status of a crop at various spatial scales. Estimation of crop yields^3,4^, evapotranspiration within a field^5^, or assessment of the leaf chlorophyll or carotenoids contents^6–8^ are examples of such use.

Remotely-sensed data are also used for detecting plant foliar diseases at various spatial scales and with different sensors and methods^9–11^. Methods developed for leaf disease detection are usually based on empirical relationships between spectral indices and the presence or degrees of intensity of the disease. Spectral indices can be computed easily and efficiently, require a limited amount of spectral information, and usually provide indirect information on the presence of the disease by capturing the effects of symptoms (such as chlorosis) on the leaf optical properties. Gennaro *et al*.^12^ used unmanned aerial vehicles (UAVs) to acquire very high spatial resolution data of vineyards fields, from which leaves contaminated with grapevine leaf stripe disease (*Phaeomoniella chlamydospora*) could be discriminated from healthy leaves based on Normalized Difference Vegetation Index (NDVI) values. However, the authors outlined that such a method is only reliable if no other factors affect leaf chlorophyll. Indeed, as various biotic and abiotic stresses may affect leaf chlorophyll content (e.g., nitrogen stress, pests, etc.), the NDVI cannot discriminate a specific disease from other stresses. Different methods have been tested to overcome this limitation. Lu *et al*.^13^ used a non-imaging spectrometer to compute spectral vegetation indices and discriminate various tomato leaf diseases using a K-nearest neighbour classifier. Although the method showed promising results, it was limited by the difficulty to specifically measure the reflectance on the disease spots, creating mixed healthy and infected spectra. Mahlein *et al*.^14^ used hyperspectral signatures of healthy and infected sugar beet leaves acquired in laboratory conditions with a non-imaging spectrometer to develop specific disease indices. However, such indices may be affected by directional effects induced by the leaf surface, and the obtained empirical relationships may show moderate extrapolative abilities, e.g., when applied to other crops or different sensors.

Another widely used method to detect foliar diseases based on remote-sensing data relies on the development of multivariate regression models based on the whole reflectance spectrum. Such methods allow the full spectral information contained in the visible and near infrared (VNIR) and/or the shortwave infrared spectral range (SWIR) to be taken into account, instead of only a limited number of spectral bands, as when using spectral indices. Delalieux *et al*.^15^ acquired hyperspectral data of healthy and *Venturia inaequalis*-infected apple leaves with a non-imaging spectroradiometer, and compared several multivariate statistical approaches. They showed that the SWIR range contains relevant information for the early discrimination of healthy and infected leaves. Yeh *et al*.^16^ used hyperspectral images acquired in a laboratory on healthy and *Colletotrichum gloeosporioides*-infected strawberry leaves, and compared various multivariate methods for discriminating between healthy and infected leaves. Their results showed that it was possible to select optimal wavelengths in the VNIR range in order to discriminate healthy leaves from those contaminated at incubation (i.e., early) and symptomatic stages.

Although multivariate regression models are powerful tools for the detection of plant diseases, these techniques are limited by the exhaustiveness of the database used to calibrate the models. Furthermore, similarly to spectral indices, such models remain potentially sensitive to light conditions and vegetation structure at various scales, including leaf geometry. As a consequence, statistical models derived from spectral indices or full spectral analyses may show limited generalisation abilities, because of the multiple factors influencing reflectance that must be taken into account when producing a training dataset. Alternatively, physically-based models are promising tools to overcome these limitations, since their inversions allow disentangling the influences of multiple factors on spectral information, including leaf biochemistry, leaf and canopy structures, and acquisition conditions. Radiative transfer models (RTM) describe the interactions between light and matter. In the case of vegetation, these interactions occur from molecular scale, due to the absorption of photons by leaf biochemical constituents such as pigments, to larger scales, due to the scattering of photons within the leaf and canopy internal structures. Although RTMs are designed to simulate the interaction of light with healthy and senescent leaves, some authors used such models for the detection of foliar diseases. Caldéron *et al*.^17^ used the PROSAIL model^18–20^ as a validation tool to study the effects of varying chlorophyll content and leaf area index on simulated spectral indices, including NDVI and the R_550_/R_670_ ratio. Those simulated indices were compared with multispectral images acquired with UAVs in poppy fields contaminated with downy mildew (*Peronospora arborescens*). Albetis *et al*.^21^ used multispectral images acquired with a UAV to detect *flavescence dorée* disease (*Candidatus Phytoplasma*) in vineyards. Their results showed that, in the case of red cultivars, the anthocyanin content derived from PROSAIL inversion is a relevant proxy for the discrimination of *flavescence dorée* symptomatic leaves against the asymptomatic ones. However, to our knowledge, there have been no previous attempts to study the impact of a disease on leaf structure and biochemistry based on physical modelling applied to remote sensing. Such knowledge would help to better understand the interactions between the leaf host and the pathogen, which could then be used to develop methods for, e.g., early disease detection (i.e., when symptoms are still not visible to the naked eye) or for discriminating a disease-induced stress from other biotic or abiotic stresses.

In this paper, we used PROSPECT-D^22^ combined with COSINE^23^ (PROCOSINE) in order to estimate leaf biochemical and structural parameters from close-range imaging spectroscopy of banana leaves, and to identify the degree of infection from black leaf streak disease (hereinafter referred to as BLSD) a foliar disease caused by the Dothideomycete fungus *Pseudocercospora fijiensis* (previously *Mycosphaerella fijiensis*). BLSD is a major disease affecting bananas^24^, first identified in the Sigatoka Valley in Fiji in 1963^25^. It causes leaf necrotic lesions that eventually lead to (1) a yield reduction of up to 50%, due to the reduction of photosynthesis^26,27^, and (2) a premature ripening of bunches, making the bananas inappropriate for export and commercialisation^28,29^. The objective of this exploratory study was to assess the potential of the combined PROCOSINE model to (i) monitor the changes in leaf structure and biochemistry caused by the development of the disease and (ii) identify the degree of infection as estimated by expert knowledge. Indeed, discriminating early infection stages from the later ones can help to decide on the protection strategy to adopt, i.e., preventive (by using contact fungicides) for early stages or curative (by using systemic fungicides) for later stages. Disks of banana leaves at different stages of infection from *Pseudocercospora fijiensis* were imaged in laboratory conditions with a VNIR hyperspectral camera. Biochemical and biophysical foliar properties, including chlorophyll (*C*_*ab*_), carotenoids (*C*_*cx*_), anthocyanins (*C*_*ant*_), and brown pigments (*C*_*bp*_) contents, structure parameter (*N*), light incident angle (*θ*_*i*_) and specular reflection (*b*_*spec*_) were then estimated by model inversion on a pixel basis, leading to submillimeter maps of PROCOSINE parameters. Based on these maps, the effect of BLSD on the ecophysiology of the leaves was studied. Finally, a linear discriminant analysis was performed on the inverted parameters in order to discriminate the disease stages.

## Results

### Influence of the disease on leaf reflectance

**Figure 1:**
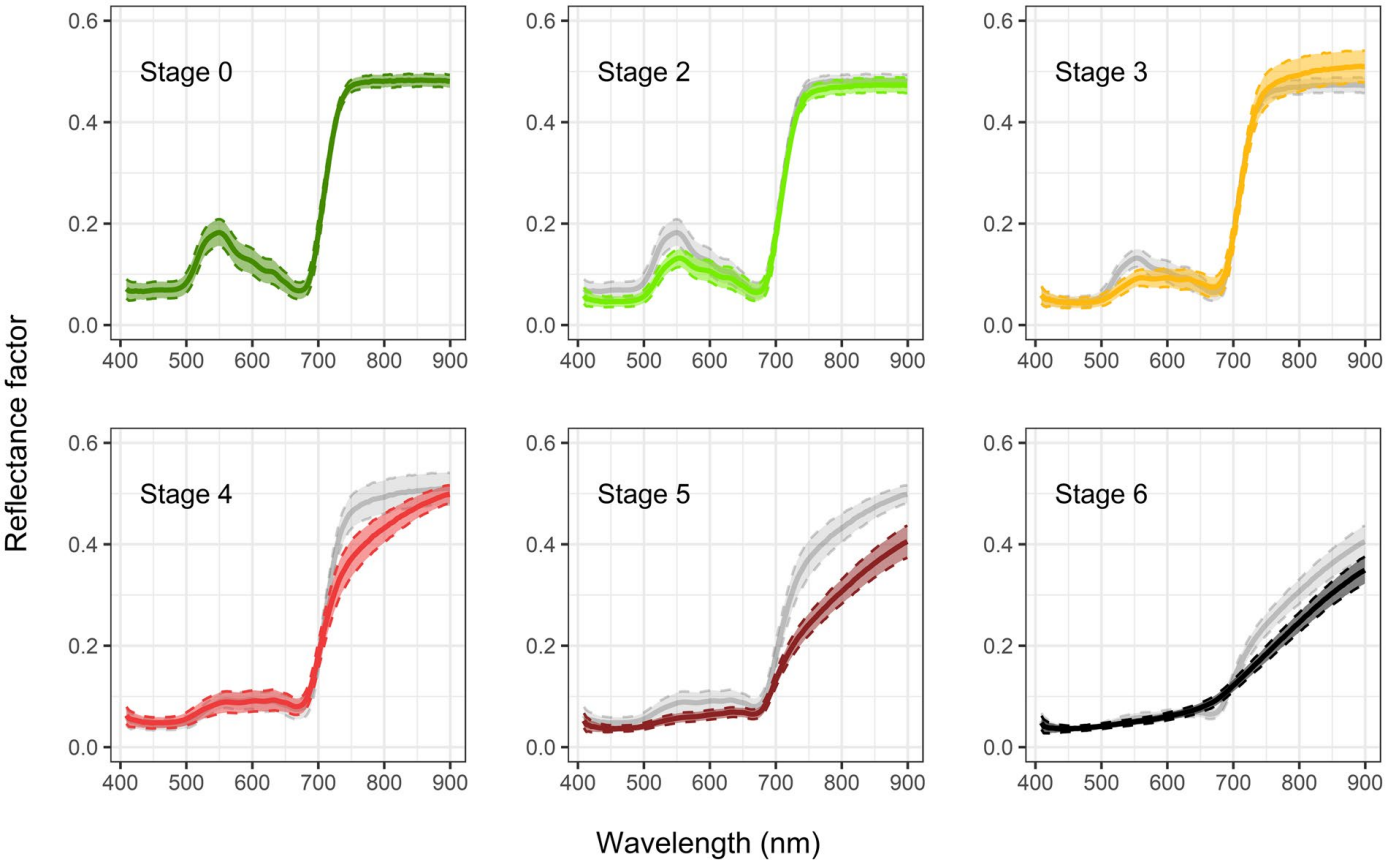
Mean reflectance spectra (solid lines) and standard deviation (dashed lines) of asymptomatic and BLSD-infected leaf tissues at different stages of the disease (stage 0 to 6). Grey spectra show the reflectance values for the previous disease stage.

The mean reflectance spectra of asymptomatic and BLSD-infected leaf tissues were first computed based on pixels extracted from hyperspectral images (Fig. 1).

The reflectance spectra corresponding to asymptomatic pixels were consistent with our expectations: in the VIS domain, the reflectance was minimum in the blue and red spectral domains, and maximum in the green spectral domain. It showed a sharp increase from 680 nm to 740 nm (the so-called red edge), before reaching a plateau in the NIR domain (740 nm to 900 nm).

Overall, the reflectance decreased in the VIS domain from 400 nm to 650 nm with the development of *P. fijiensis*. This decrease was more pronounced in the green spectral domain (around 550 nm) than in the blue (around 450 nm) and red (around 650 nm) ones. A slight increase in reflectance associated with the presence of the disease was observed in the domain from 650 nm to 700 nm. For longer wavelengths and towards the NIR domain, *P. fijiensis* strongly impacted the measured reflectance, as the sharp shoulder of the red edge around 750 nm observed for asymptomatic leaves tended to smoothen with increasing disease stage, and completely disappeared for stages 5 and 6 leaves. In the NIR domain, the reflectance showed a slight increase for stage 3, and a more pronounced decrease for stages 4, 5 and 6.

### Dynamics of the inverted parameters

**Figure 2:**
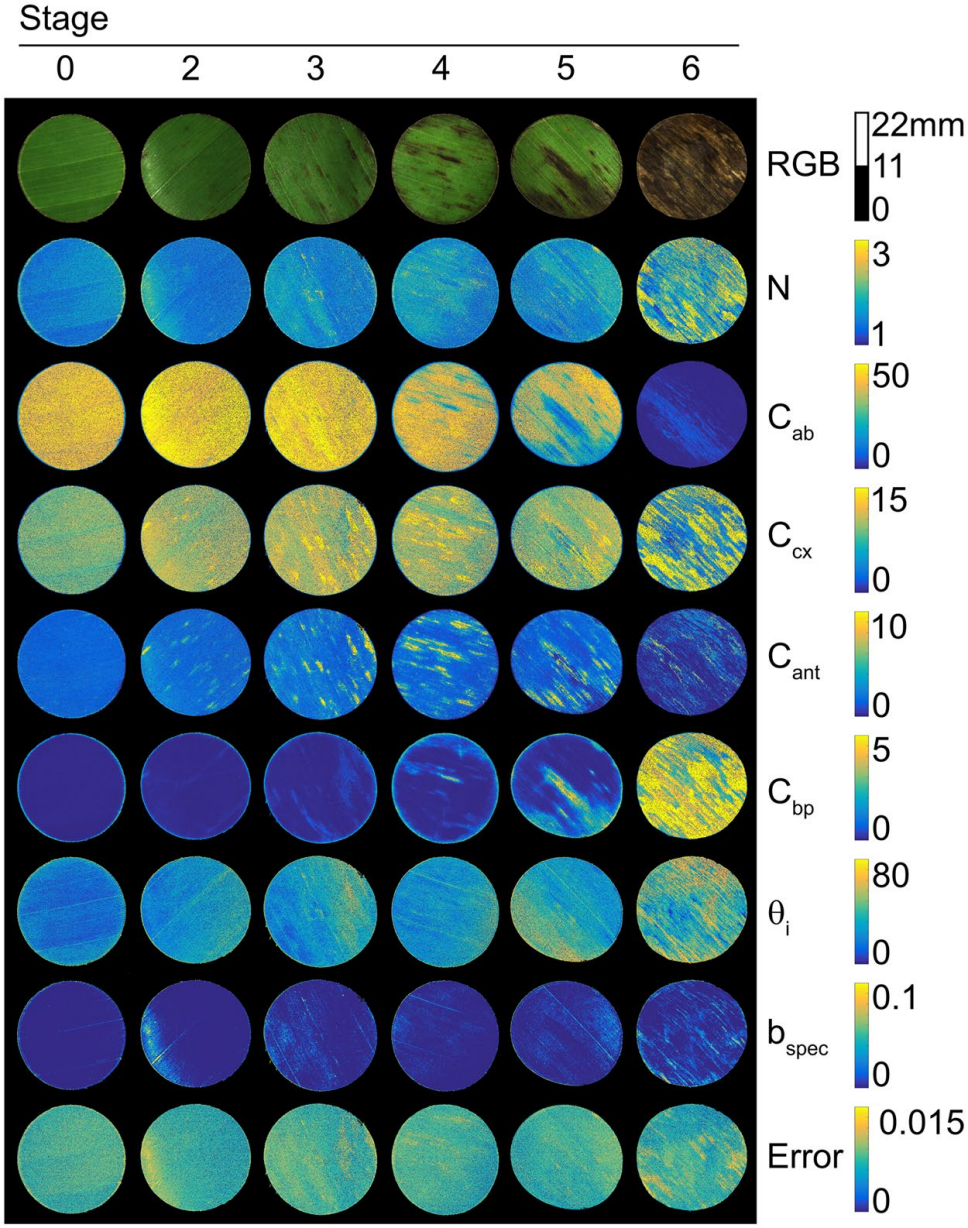
Examples of inverted PROCOSINE parameter maps. “RGB” are visualizations of the hyperspectral images of the adaxial side of the leaf disk. *N* and *b*_*spec*_ are unitless, *C*_*ab*_, *C*_*cx*_,*C*_*ant*_ are expressed in *μg*.*cm*^−2^, *C*_*bp*_ is given in arbitrary units and *θ*_*i*_ is expressed in degrees. “Error” indicates the reconstruction error maps, expressed in reflectance factor (unitless). The scale next to the RGB images indicates the size of the leaf disks.

PROCOSINE was then inverted for each pixel of the image database. The estimated PROCOSINE parameters were then used to compute simulated spectra, from which was computed the reconstruction error. Example maps of inverted parameters and reconstruction errors are presented in Fig. 2 for illustrative purposes. Note that the results obtained for *C*_*w*_ and *C*_*m*_ are not shown here, due to their limited influence on reflectance in the VNIR domain. Error spectra as a function of the wavelength are also presented in Supplementary Fig. 1.

BLSD spots did not appear on every map of inverted parameters. *N*, *θ*_*i*_ and *b*_*spec*_ did not show any clear visible variation on infected areas. *C*_*ab*_ showed lower values on infected areas, while *C*_*bp*_ and, to a lesser extent, *C*_*cx*_ values tended to increase on infected areas. *C*_*ant*_ increased until stage 4, to finally decrease with stages 5 and 6. Low values (less than 0.015) obtained for reconstruction errors indicate that the modelled spectra were consistent with measured ones. Although stage 6 showed a slight increase in reconstruction error on circumscribed areas, the precision of the inversion of PROCOSINE was not notably affected by the disease. Finally, the error spectra also showed low values (Supplementary Fig. 1), although patterns were observed for different spectral regions. The blue region (400 to 500 nm) of the spectrum showed a relatively high standard deviation. The shape of the error spectra in the range of about 500–650 nm changed from oscillating at high frequency in stage 0 to lower frequency and smoother oscillation pattern in the later stages. Around the red edge, the error increased between stage 0 and stage 3 and decreased again.

**Figure 3:**
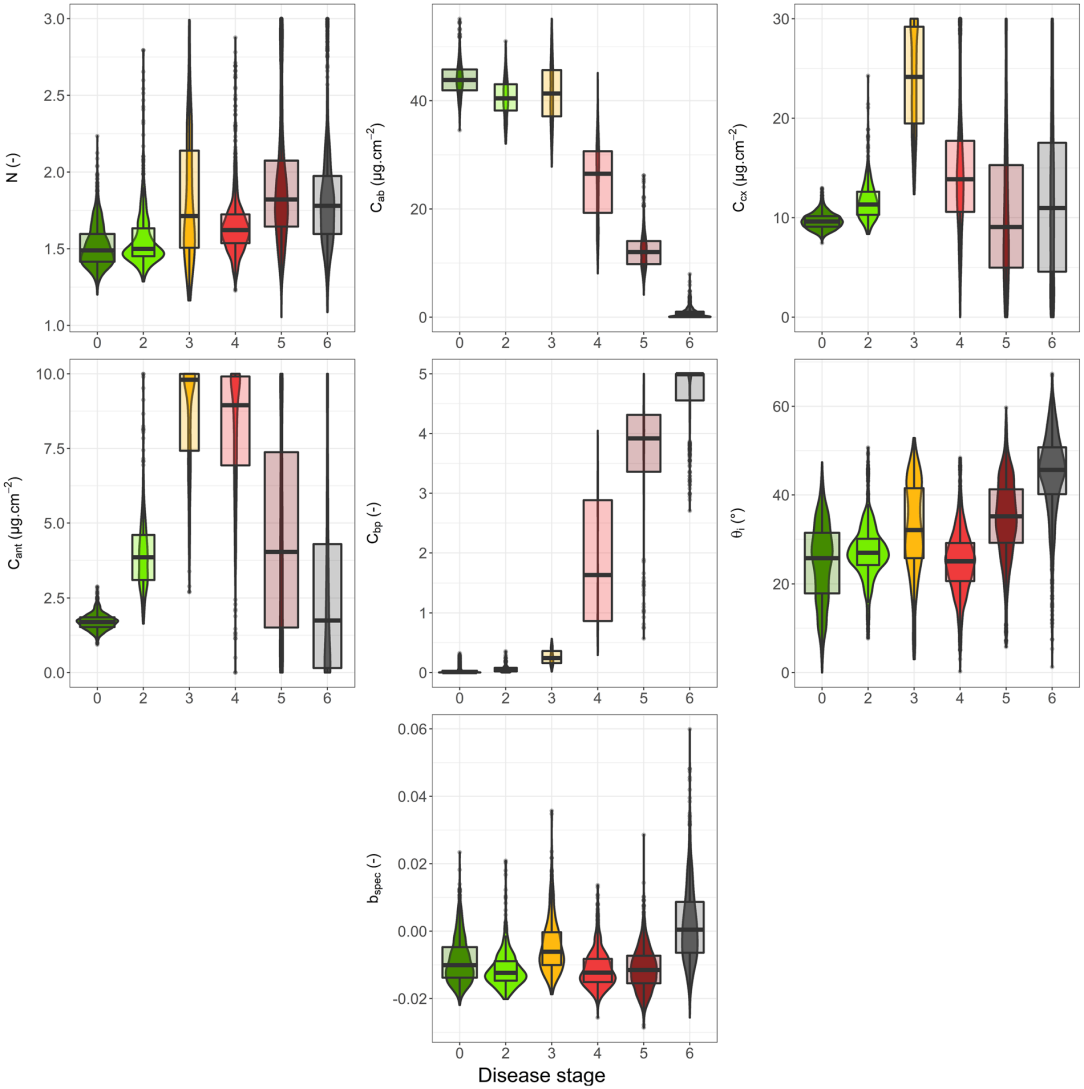
Boxplots (outliers, minimum, first quartile, median, third quartile and maximum) and distribution of estimated PROCOSINE parameters according to the stage of black leaf streak disease. The width of the boxplots indicates the number of samples for each group. This number ranges between 445 (stage 3) and 1504 (stage 0).

Boxplots and distributions of the complete pixel dataset were computed to monitor the dynamic of each PROCOSINE parameter according to the disease stage (Fig. 3).

The mesophyll structure parameter (*N*) dynamic showed a slight increase as the disease spreads, ranging from a median value of 1.52 at stage 0 (asymptomatic stage) to 1.82 at stage 5. An increase of the dispersion of the values for stages 3, 5 and 6 is also evident. *C*_*ab*_ showed a decrease starting between stages 3 and 4, and median values ranged from 43.90 µg.cm^−2^ for asymptomatic leaves, to 0.69 µg.cm^−2^ for stage 6. *C*_*cx*_ increased between stages 0 to 3, decreased between stages 3 and 5, and finally stabilised for stages 5 and 6. Median values ranged between 9.06 and 24.15 µg.cm^−2^, and a strong dispersion was found for stages 3, 4, 5 and 6. *C*_*ant*_ displayed a bell-shaped pattern, i.e., it increased between asymptomatic stage and stage 3, before decreasing from stage 4 to stage 6. Median values ranged from 1.70 to 9.80 µg.cm^−2^, with a strong dispersion for all stages, excepted for asymptomatic stage. *C*_*bp*_ displayed low values from asymptomatic stage to stage 3, then dramatically increased at stage 4, with median values ranging from 0.01 to 4.99. Dispersion was also very pronounced for stages 4, 5 and 6. *θ*_*i*_ displayed an increase after stage 4. In particular, the values for stage 6 were higher than those obtained for other stages (median values ranging from 24 to 44°), with a strong dispersion of *θ*_*i*_ values for all disease stages. Finally, there was no clear dynamic for the specular term *b*_*spec*_, although stage 6 exhibited slightly higher values and an increased dispersion. The median *b*_*spec*_ values ranged between −0.01 and 0.00.

### Discrimination of disease stages

**Figure 4:**
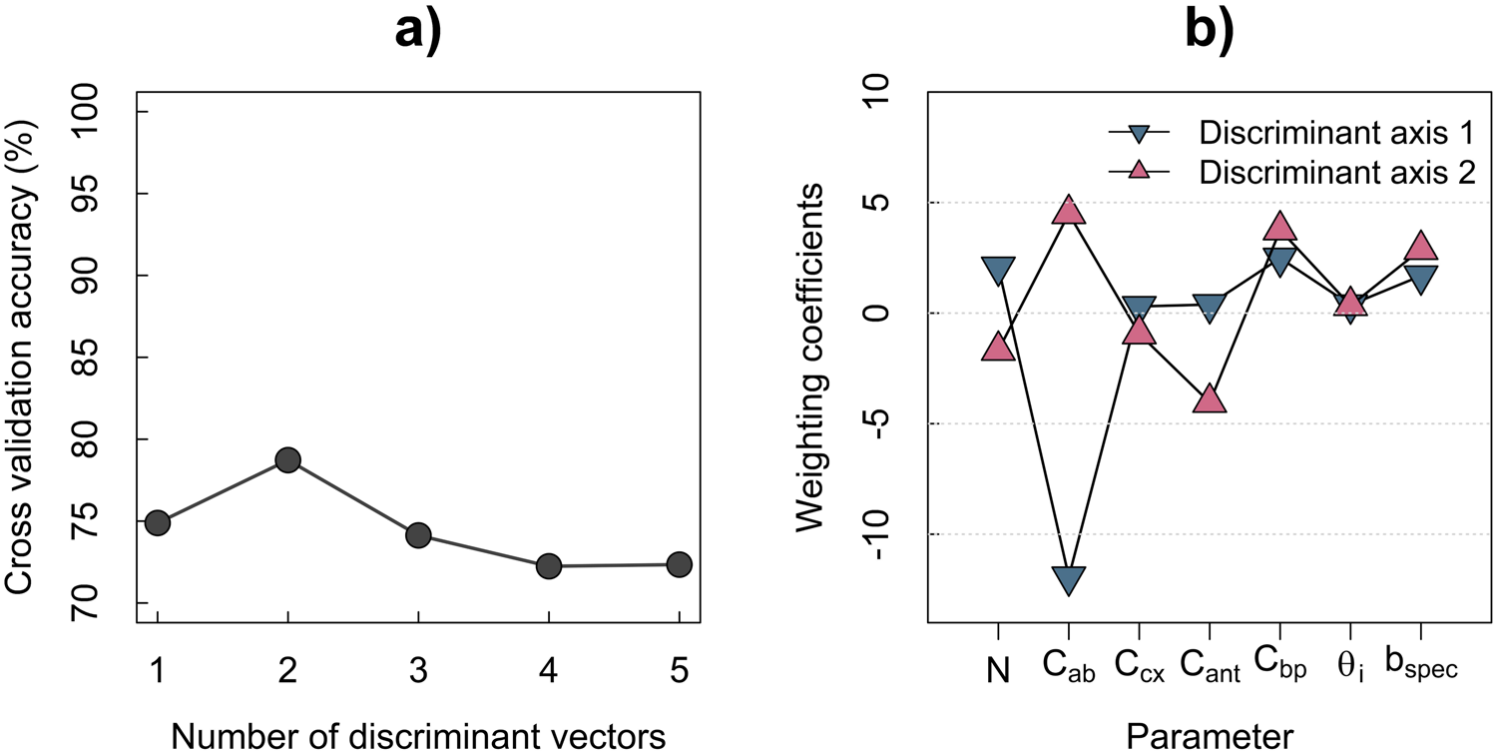
Results of the linear discriminant function analysis (a: overall classification accuracy obtained using two-fold cross-validation as a function of the number of discriminant axes; b: weighting coefficients of PROCOSINE parameters obtained for the first two discriminant axes and computed over the complete data set).

A two-fold cross validation linear discriminant analysis (hereinafter referred to as LDA) was used to classify the different disease stages based on the results of PROCOSINE’s inversion. The overall accuracy of the model was then computed for a variable number of discriminant axes. Figure 4a shows that the first two axes provided optimal performances, with 78.7% of correct classifications, while avoiding overfitting. The confusion matrix associated with these classification results provides more insights into the classifier performance (Table 1). The best performance was achieved for the discrimination of stage 5 (producer accuracy: 95.7%). The identification of stages 2 and 4 pixels was more difficult (producer accuracies of 61.8 and 50.3%, respectively) due to confusions with stage 0 and 3, respectively.

**Table 1:** Confusion matrix computed from the LDA classification results obtained using two-fold cross-validation.

		Expert knowledge						User accuracy
		0	2	3	4	5	6	
LDA results	0	1218	168	15	0	0	0	86.9
	2	286	381	89	28	0	0	48.6
	3	0	65	307	172	0	0	56.4
	4	0	3	34	504	26	0	88.9
	5	0	0	0	299	1344	45	79.6
	6	0	0	0	0	34	923	96.5
	Producer accuracy	81.0	61.8	69.0	50.2	95.7	95.4	**78.7**

**Figure 5:**
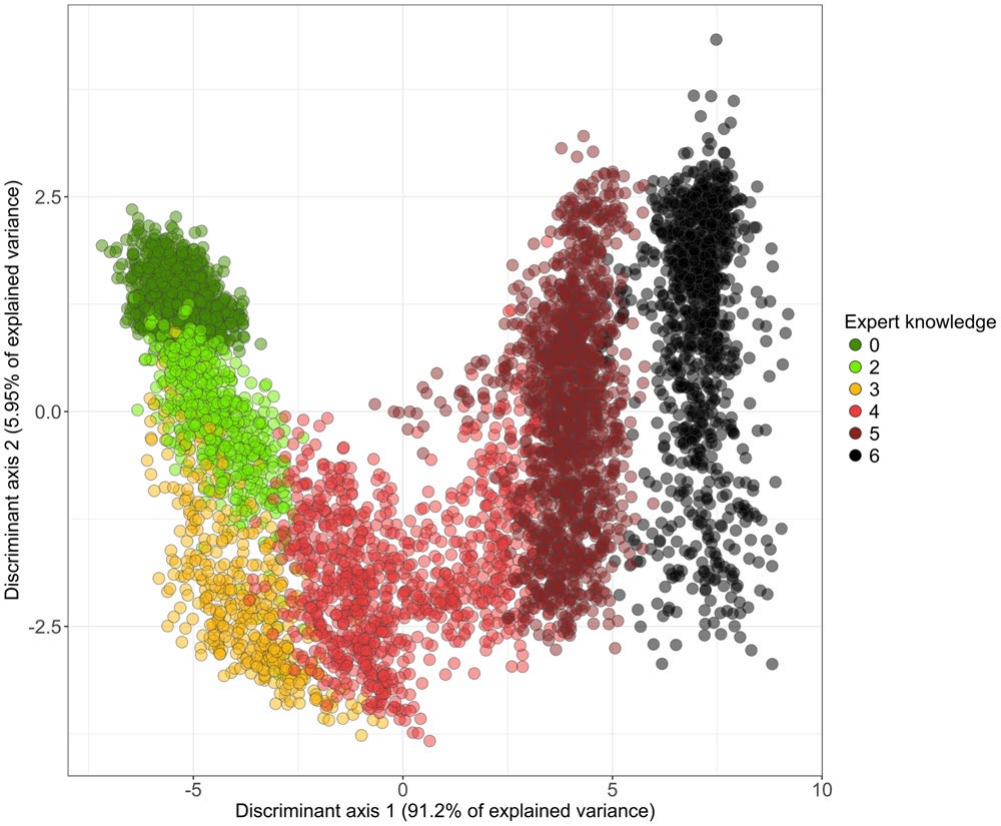
Sample distribution in the LDA plane defined by the first two linear discriminant vectors computed on the complete data set.

A new LDA model was then calibrated using the complete dataset to compute the weighting coefficients of the parameters used to build the discriminant vectors. With a weighting coefficient value three-times that of the other ones on the first discriminant axis, *C*_*ab*_ was the main parameter for the classification of the disease stages. *C*_*bp*_ and *C*_*ant*_ also appeared to carry critical information, with coefficients higher than 3 in absolute value for the second axis (Fig. 4b). Figure 5 shows that the first axis mainly discriminated stages 3, 4, 5 and 6, with some overlapping between stages 3 and 4. The second axis discriminated the asymptomatic stage from stages 2 and 3, again with some overlapping between stages 0 and 2.

## Discussion

Pixels belonging to stage 0 show reflectance features characteristic of healthy green vegetation^18,30,31^. We observed a global decrease of reflectance in the VIS and NIR spectral domains as the disease spreads, except for the 650–700 nm range (Fig. 1). The results obtained from model inversion (Figs 2 and 3) suggest that the associated decrease in reflectance may result from an increase in *C*_*cx*_ and *C*_*ant*_, as observed in various situations of stress^32,33^. An increase in *C*_*cx*_ and *C*_*ant*_ is physically sound to explain the decrease of reflectance for the 500–650 nm range, as it shows unsaturating reflectance for the asymptomatic stage. However, considering the saturating reflectance (i.e., that does not change with a further increase in absorption) for the blue (400–500 nm) range, the observed decrease is more likely to be due to changes in surface properties unaccounted for by the model. The slight increase of reflectance in the NIR domain for stage 3 can be explained by disorganisation of the structure of the mesophyll. The strong decrease of reflectance measured in the NIR domain that starts from stage 4 can be explained by the increasing amount of brown pigments or other denatured proteins absorbing the light below 1300 nm^34,35^, as confirmed by the results of model inversion.

The increase of reflectance in the 650–700 nm range is explained by a decrease in *C*_*ab*_ which leads to decreased absorption. For stages 4, 5 and 6, the decrease in absorption due to the reduction of *C*_*ab*_ is compensated by the increased amount of brown pigments that also absorb light in this spectral domain. *N* shows a slight increase as the disease spreads. This parameter describes the number of theoretical homogeneous layers composing the mesophyll, and can be considered a measurement of the optical complexity of the inner leaf medium. The increase of *N* can be linked to the action of the fungus on the leaf. Indeed, *P. fijiensis* is an hemibiotrophic fungus^36^, with first a biotrophic phase which only colonizes the intercellular spaces between mesophyll cells, hence initially having little influence on the global structure of the mesophyll. However, this first step is followed by growth in all intercellular mesophyll layers and air chambers, then by growth of conidiophores through the stomata and then the first alterations of mesophyll cells are observed (at the beginning of the necrotrophic phase), which may explain the slight disorganisation of the vegetative medium^37^. The response of *C*_*ab*_ is also consistent with expectations, as the development of *P. fijiensis* induces chlorosis^36^. *C*_*ab*_ starts decreasing beyond stage 3, suggesting that *C*_*ab*_ is not significantly affected at the first disease stages. *C*_*cx*_ tends to increase at the first stages of disease, more particularly between stage 2 and 3 (Fig. 3). Many studies have reported an increase of the carotenoids content as a response to various environmental stresses^33,38–40^. However, to the best of our knowledge, there have been no reports of *C*_*cx*_ increases induced by pathogen-linked specific stresses. The dynamic of *C*_*cx*_ can also be explained by other factors unaccounted for by PROCOSINE, including biochemical constituents produced by the fungus or the immune system of the plant, or changes in leaf surface properties or internal structure. As illustrated in Fig. 3, *C*_*ant*_ increases from stage 0 to 3, before decreasing from stage 4 to 6. The increase in *C*_*ant*_ observed during the early stages of infection is also visible in Fig. 2. Interestingly, these results are in agreement with previous studies that reported an increase in *C*_*ant*_ as a response to different types of stresses^32,41^, including the appearance of pathogens in vegetative tissues^42^ using direct biochemical measurements. The increase in *C*_*bp*_ is consistent with expectations, as brown pigments appear during senescence and diseases, with complexing of oxidized phenols^43,44^. Brown pigments are absent in healthy leaves or during the first stages of the infection. The slight increase observed for *θ*_*i*_ might be explained both by the disorganisation of the medium while the fungus grows and spreads, and by the progressive evaporation of the cell-contained water, leading eventually to the collapse of the cell. This might also come from compensations between *C*_*bp*_ and *θ*_*i*_ as observed for necrotic tissues by Jay *et al*.^23^. The dynamic of the specular reflection *b*_*spec*_ shows no particular trends, which suggests that this parameter is not affected by the development of *P. fijiensis*, or that the current parameterisation of the model or the spatial resolution used in this study does not take into account potential variations in *b*_*spec*_.

The differences between simulated and measured spectra (supplementary Fig. 1) can be explained by different factors according to the spectral range: in the blue domain, the error probably comes from the inherent noise of the sensor at such wavelengths. The variations with high frequencies observed for the mean error in the 500–750 nm range may be explained by a slight shift of the spectral calibration of the sensor. Indeed, the differences between measured and simulated spectra tend to become more important when occurring in spectral regions with rapid changes of reflectance, such as for the green or red edge regions. The concomitant reduction of those differences when reaching late stages of the disease would support this hypothesis, with the observed slopes of the green and red edge regions decreasing. Finally, the variations with low frequencies observed in the visible and the NIR regions for stage 3 and later stages might come from the fact that the pathogen is pushing the model toward the limits of its domain of validity, especially with the potential appearance of new chemical compounds that are not taken into account by PROSPECT.

**Figure 6:**
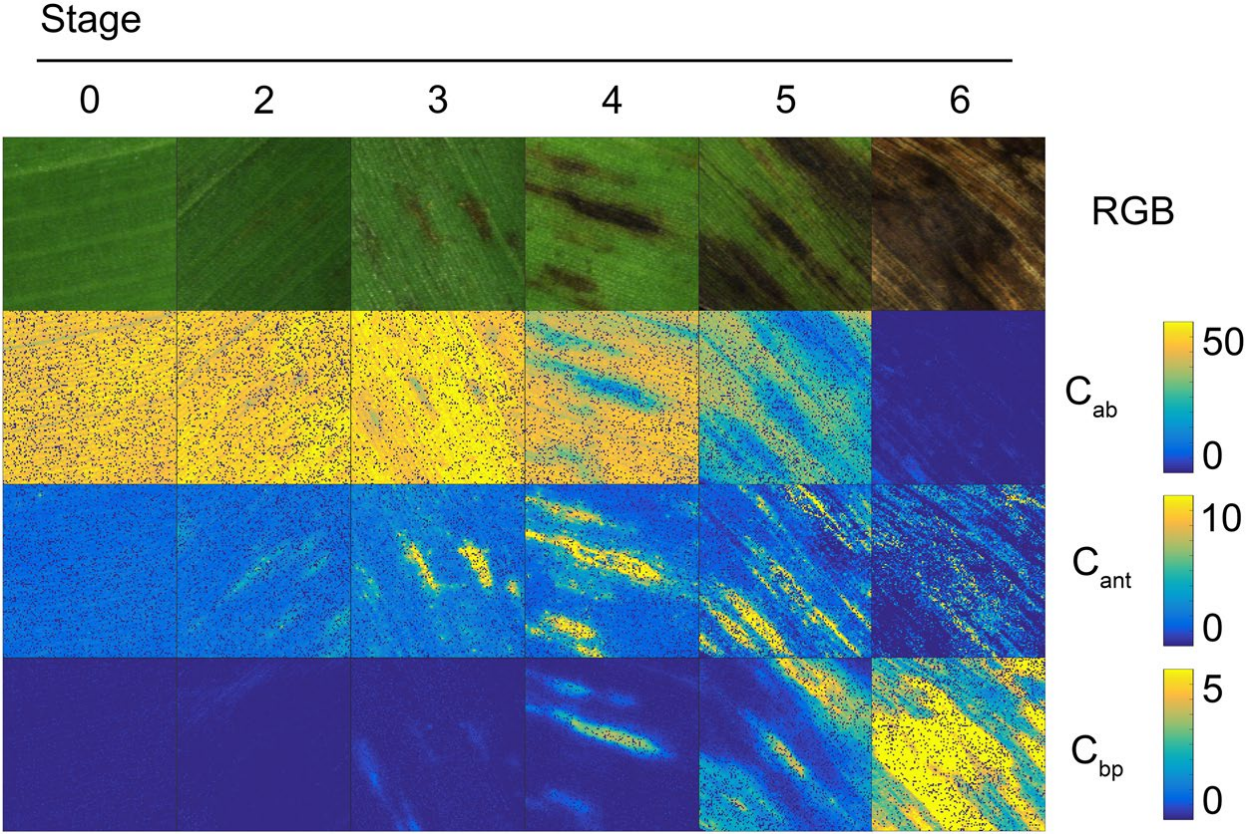
Interactions between *P. fijiensis* and the most sensitive PROCOSINE parameters according to the disease stage. *C*_*ab*_ and *C*_*ant*_ are expressed in *μg*.*cm*^−2^, *C*_*bp*_ is given in arbitrary units.

The discrimination of stages 3, 4, 5 and 6 is based on the first linear discriminant axis (Fig. 5), with *C*_*ab*_ as the most important variable for this axis (Fig. 4b). The high accuracy achieved for the classification of stages 5 and 6 is consistent with the results presented in Fig. 2 and further illustrated in Fig. 6, as clear differences in chlorophyll content appear between stages 4, 5 and 6. However, confusion occurs in discriminating between stages 3 and 4. This is probably due to the fact that stages 3 and 4 are differentiated by the geometry of the disease spot, which is not taken into account here.

As mentioned earlier, the LDA results show that the first stages of the disease (i.e., stages 2 and 3) are difficult to discriminate. More particularly, confusion occurs between pixels related to stage 0 and stage 2. Figure 5 shows that the separation of stages 0 and 2 is mainly explained by the second discriminant vector. Variables that contribute the most to this axis are *C*_*ab*_, *C*_*ant*_ and *C*_*bp*_ (Fig. 4b). Among those parameters, only *C*_*ant*_ shows different values between stages 0 and 2 (Fig. 6), despite some overlap of the two distributions (Fig. 3) that might explain the poorer accuracy for discriminating stages 0 and 2. The confusion between stage 0 and 2 can also be explained by the size and spatial distribution of early stages spots (i.e., stage 2) that can lead to the inclusion of asymptomatic pixels into the stage 2 subset (see Supplementary Fig. 2). However, with an overall accuracy of 78.7%, the use of a LDA model applied to the inversion results of PROCOSINE is a relevant approach for discriminating the disease stages of *P. fijiensis*-infected banana leaves. Moreover, the increase in *C*_*ant*_ during the first stages of the spreading of the fungus makes it a promising proxy for early disease detection.

PROSPECT is a physically-based model describing the interactions between light and leaf tissues in order to simulate leaf optical properties resulting from foliar biochemical and structural properties. However, models are based on the simplification of complex systems. In the case of PROSPECT, one of the main advantages of the model is its straightforwardness: it does not seek to exhaustively describe the complexity of the leaf, and simplifications such as its generic refractive index and the simplistic description of the leaf/air interface are well known^22,45^. When considering the physiological impact of foliar diseases on leaf tissues, it is possible that the unusual plant responses to the disease development may be beyond the original domain of validity of PROSPECT, and lead to “exotic combinations” of parameters. For example, PROSPECT-D only includes *C*_*ab*_, *C*_*cx*_, *C*_*ant*_ and *C*_*bp*_ to describe the absorptive effects of leaf constituents in the optical domain, and the presence of secondary pigments (e.g., flavonoids) or other optically-active chemical constituents that might be induced by the development of the fungus are not considered. Moreover, the three families of pigments accounted for by PROSPECT (i.e., chlorophylls, carotenoids and anthocyanins) also rely on assumptions. These pigment families encompass a large variety of different molecules, and the stoichiometry of these molecules is supposed to be identical among vegetation, as they are defined by a unique specific absorption coefficient. Therefore, changes in specific absorption of anthocyanins induced by the variations of pH are not taken into account^46^. Pathogens may induce changes in the pH of part or all of leaf cells, resulting in changes in the optical activity of anthocyanins, which, in our case, can be interpreted as a variation in content of *C*_*ant*_. Finally, PROSPECT is a plate model, describing the leaf as a stack of several homogeneous layers, described by the *N* parameter. The fungus, although supposed to have little impact on the inner structure of the medium, will introduce some heterogeneity while penetrating the inter-cell space and activating its defences, which may push the model toward its limits from a physical point of view. However, the error spectra remain low (Supplementary Fig. 1), and the reconstruction error (Fig. 2) do not show any clear pattern depending on the disease stage. Consequently, values derived from PROSPECT inversion can be considered as indicators of biophysical and chemical changes in leaf tissues. However, those values cannot be used as absolute values for the leaf properties included in PROSPECT, as long as no proper validation has been performed. Indeed, working at sub-millimetric scales is a prerequisite for the early detection of diseases^47^. Due to the size of the investigated areas, the experimental setup required to perform biochemical analysis on destructive samples that could be used as absolute values go beyond our exploratory study. Despite these limitations, the results shown in previous sections demonstrate that PROCOSINE (i) provides dynamics that are consistent with physiological expectations and (ii) is useful to investigate the effects of *P. fijiensis* and, more generally, of plant diseases on leaf tissues.

The exploratory results underline that the ecophysiological processes inferred from PROCOSINE inversion can help understand how banana leaves are affected by black leaf streak disease. Indeed, the first stages show no to little variations in brown pigments and chlorophyll contents, the latter being a key variable for most of the current vegetation indices. Consequently, this suggests that the classical approaches based on such vegetation indices may be not sufficiently relevant for the early detection of black leaf streak disease. Instead, particular emphasis should be paid to the use of anthocyanin-sensitive indices such as those proposed by Gitelson *et al*.^48^. One potential perspective would be to try to detect the BLSD at the field level using a drone-mounted multispectral camera with the relevant spectral bands to estimate the anthocyanins content. Another interesting prospect would be to acquire images in the SWIR range, as this would enable inclusion of the leaf dry matter and water contents, which can be interesting indicators of the development of the fungus.

More generally, the inversion of radiative transfer models such as PROCOSINE appears to be an interesting tool to investigate the effects of diseases on foliar systems from a physiological point of view. Such an approach could guide the selection of appropriate vegetation indices, e.g., for the early detection of plant diseases or to discriminate disease-related stresses from water, nitrogen or other biotic stresses. The inversion of radiative transfer models for detecting and discriminating disease stages should be further assessed with a larger dataset, including various cultivars and image acquisition conditions. This would allow a rigorous comparison between a purely statistical method (e.g., LDA) and the hybrid method used in this study (PROCOSINE and LDA), and also to confirm the current performance with an independent dataset. One issue to assess is that, depending on the disease’s type and stage, the inversion performance may be limited by the fact that the biochemical and biophysical conditions are out of the boundaries for which the model was validated (e.g., with a strong necrosis of the leaf). Further investigation should be done in order to provide proper validation and confirm or refine our analysis in the case of the influence of the pathogen *P. fijiensis* on banana leaves of different ages, from different accessions and with different level of resistance, and new tests with larger datasets should be performed to assess the robustness of the method. The use of PROCOSINE based on high spatial resolution images of plant canopies would also be an interesting prospect to explore, although the model has not been designed to properly handle the radiative transfer conditions that prevail in such situations, i.e., with a strong influence of the pixel environment.

## Conclusion

The coupled PROSPECT-D + COSINE model (PROCOSINE) was used to study the response of structure and chemistry of banana leaves when infected with black leaf streak disease (BLSD) caused by the fungus *P. fijiensis*. For this purpose, asymptomatic and BLSD-infected pixels were first extracted from laboratory acquired hyperspectral images and were given a disease stage based on expert knowledge. Foliar chemistry and structure were then retrieved based on PROCOSINE inversion for each pixel. Finally, a linear discriminant analysis was performed to estimate the disease stage of the pixels of our database based on their inverted PROCOSINE parameter values.

Our results show that most of the dynamics of estimated PROCOSINE parameters are consistent with expectations. *C*_*ab*_ showed a progressive decrease, starting at stage 4, while *C*_*bp*_ and, to a lesser extent, *N* and *θ*_*i*_ tended to increase with the spread of the disease between stages 3 and 4. *C*_*cx*_ and *C*_*ant*_ showed an interesting dynamic, increasing between stage 0 and 3, then decreasing. The dynamic of *b*_*spec*_ did not show any specific pattern. Interestingly, *C*_*ab*_, *C*_*ant*_ and *C*_*bp*_ appear to be particularly sensitive to the development of the fungus. These dynamics can be related to the action of the fungus on the leaf, for example the induced progressive chlorosis and brown pigment appearance, or the increase in anthocyanins as a response to the spreading of the disease. Furthermore, the classification results show a good overall accuracy 78.7%). The latest stages are the easiest ones to be retrieved, while more confusion occurs between stages 0, 2 and 4. Of particular interest is the anthocyanins content, which appears to be particularly sensitive to the first infection stages. Further investigation on the detection of the very early infection phase (i.e. stage 1) should be performed using intermediate values of *C*_*ant*_ and *C*_*ab*_ to those obtained for stages 0 and 2.

## Methods

### Plant material and reference measurements of disease stage

Five symptomatic and asymptomatic banana leaves (*Musa* AAA cv. Williams, Cavendish subgroup) were used in this study. All leaves were contaminated by natural inoculation and harvested after the appearance of symptoms of BLSD caused by the fungus *Pseudocercospora fijiensis*. Observed symptoms ranged from stage 2, corresponding generally to a brown strip on the adaxial surface of the leaf, to stage 6 (necrotic leaf tissue) with grey dry spots surrounded by a black ring as described by Fouré^49^. No stage 1 BLSD spots were used in this study due to the difficulty for experts to identify it, as it is only visible on the abaxial side of the leaf^49^. A total of twelve disks corresponding to various stages of infection and possibly including several disease stages (Fig. 2), were cut off at one time from leaves using a cork borer (22-mm diameter) and imaged the same day. Two disks obtained from different leaves were used for each disease stage (including the asymptomatic stage), and based on expert identification, one area of a specific stage was selected on each disk (Supplementary Fig. 2). In total, the twelve areas comprised 5941 pixels.

### Reflectance measurements

The adaxial side of leaf disks were imaged in horizontal position using a HySpex VNIR-1600 hyperspectral camera (Norsk Elektro Optikk, Norway). This camera was used to sample the reflected radiation in 160 bands ranging from 415 to 994 nm, with a 3.7 nm spectral sampling interval and a 4.5 nm spectral resolution. The camera was placed at 30 cm above the samples and acquired successive scans of 1600 pixels, with a resulting spatial resolution of approximately 0.07 mm. A halogen lamp equipped with a converging lens in order to focus the light beam on the viewing zone of the sensor was used to illuminate the sample with an incident angle of 40° relatively to the vertical direction. A 50% diffuse reflectance reference panel (Spectralon®, Labsphere) was placed horizontally at the same distance from the sensor as the leaves to estimate the incoming irradiance while limiting possible saturation of the sensor. For each pixel and each band, the bidirectional reflectance factor (BRF)^50^ was computed by dividing the signal measured over the target by that measured over the reference panel and multiplying by the reflectance value provided by Labsphere (assuming the panel to be Lambertian). Eventually, the image background was removed by image processing in order to keep only vegetation-related pixels for further analysis.

### Model inversion

The coupled models PROSPECT-D^22^ and COSINE^23^ were used to estimate the structure and chemistry of the leaves. PROSPECT is a generalisation of the “plate model” proposed by Allen *et al*.^51^, and simulates the leaf directional-hemispherical reflectance and transmittance^50^ in the optical domain ranging from 400 to 2500 nm, based on a limited number of input parameters^20^. In this study we used PROSPECT-D^22^, which is the latest release of the model. This version includes a structure parameter linked to the foliar anatomy, usually named *N*, that corresponds to the number of homogeneous layers stacked in order to represent the leaf, separated by *N*−1 air spaces. *N* is unitless and assumed to vary between 1 and 3 for the majority of non-senescent leaves^20^. Low *N* values (close to 1) correspond to leaves with a relatively simple anatomy such as monocotyledons or juveniles, while higher *N* values correspond to more complex leaf structures characteristic of dicotyledons, including differentiated parenchyma and intercellular gaps that increase multiple scattering of the light within the leaf. PROSPECT-D also simulates the effects of the three main families of foliar pigments on leaf optical properties, i.e., chlorophylls (*C*_*ab*_), carotenoids (*C*_*cx*_) and anthocyanins (*C*_*ant*_), whose contents are expressed in *μg*.*cm*^−2^. These constituents are optically-active in the visible (VIS) domain and are expected to have a particularly strong influence on our reflectance measurements. Brown pigments content (*C*_*bp*_, arbitrary unit per surface unit) is another input parameter of PROSPECT-D. It corresponds to the complexing of oxidized phenols with proteins during senescent or disease stages of the leaf, although their synthesis is not fully clarified^44,52^. Due to the optical activity of brown pigments in the VNIR spectral domain, we also expect this constituent to contribute to the banana leaf reflectance at the latest stages of the disease. Finally, the two remaining input parameters of PROSPECT-D are the equivalent water thickness (*C*_*w*_), expressed in cm, and the dry matter content (*C*_*m*_), expressed in *g*.*cm*^−2^. The ability of PROSPECT-D to simulate leaf optical properties in direct mode, and to accurately estimate leaf chemistry in inverse mode, has been validated for a wide range of species and phenology, from juvenile to senescent stages^22^.

Close-range imaging spectroscopy allows the retrieval of the pseudo bidirectional reflectance factor^23^, which means that PROSPECT cannot be used directly to simulate such spectral data and to retrieve leaf biochemistry based on model inversion. Indeed, the anisotropic effects induced by leaf surface properties (including the presence of waxes and trichomes) do not allow considering the leaf as an isotropic surface, and require taking into account leaf orientation and specularly-reflected radiation^53^. To overcome this limitation, Jay *et al*.^23^ developed the COSINE (ClOse-range Spectral ImagiNg of lEaves) model that estimates the pseudo BRF from the directional-hemispherical reflectance factor simulated by PROSPECT. The coupling of PROSPECT and COSINE (hereinafter referred to as PROCOSINE) thus relates the leaf pseudo BRF to the leaf structure and biochemistry. COSINE requires two specific parameters to compute the pseudo BRF from the reflectance simulated by PROSPECT: the light incident angle (*θ*_*i*_), i.e., the angle between the light source and the normal to the leaf, expressed in degrees, and the specular term (*b*_*spec*_) which describes the amount of specularly-reflected radiation (arbitrary unit).

PROCOSINE can be used either in forward or in inverse mode. When used in inverse mode, part or all of the input parameters are inferred from the measured reflectance factor. Various methods can be used in order to infer these input parameters, including multivariate statistical analysis, machine learning, look-up tables or iterative optimization^54–56^. In our study, iterative optimization was used in order to find the optimal combination of input parameters Θ that minimizes the merit function *J*. The latter relates the modelled and measured reflectance factors and is here given by the spectral mean squared error defined as

1 $$J({\Theta })=\sum _{\lambda =1}^{{n}_{\lambda }}\,{({R}_{meas,\lambda }-{R}_{mod,\lambda }({\Theta }))}^{2}$$

where *n*_*λ*_ is the number of spectral bands, *R*_*meas*,*λ*_ and *R*_*mod*,*λ*_ are, respectively, the measured and modelled reflectance factors at waveband *λ*, and *Θ* is the [9 × 1] vector of PROCOSINE parameters to be estimated. Among all the numerical methods available to perform iterative optimization and to estimate leaf properties from PROCOSINE inversion, we selected the trust-region reflective algorithm implemented within the *lsqcurvefit* function of MATLAB (version 8.6.0, The MathWorks Inc., Natick, MA, 2015), and applied it for each leaf pixel of the hyperspectral images. An iterative optimization was performed over the 415–900 nm spectral range, after removing the longer wavelengths because of the high noise level in this domain (using these wavebands for model inversion actually did not improve the results, not shown). For each model input parameter to be estimated, the initial value as well as the lower and upper bounds are given in Table 2. Finally, the reconstruction error was computed as the root mean squared error between measured and modelled spectra:

2 $$RMSE=\sqrt{\frac{{\sum }_{\lambda =1}^{{n}_{\lambda }}\,{({R}_{meas,\lambda }-{R}_{mod,\lambda })}^{2}}{n}}$$

where *n* is the number of samples, *n*_*λ*_ is the number of spectral bands, *R*_*meas*,*λ*_ is the measured reflectance factor and *R*_*mod*,*λ*_ the modelled reflectance factor at the *λ*^*th*^ waveband.

**Table 2:** Initial values, lower and upper bounds used for PROCOSINE inversion. *a*.*u*.: arbitrary unit. —: unitless parameter.

Parameter	*N*	*C*_*ab*_	*C*_*cx*_	*C*_*ant*_	*C*_*bp*_	*C*_*w*_	*C*_*m*_	*θ*_*i*_	*b*_*spec*_
Units	—	*μg*.*cm*^−2^	*μg*.*cm*^−2^	*μg*.*cm*^−2^	*a*.*u*.	*cm*	*g*.*cm*^−2^	°	—
Initial value	1.5	50	10	1	0.5	0.002	0.002	20	0
Lower bound	1	0	0	0	0	0.0005	0.001	0	−0.2
Upper bound	3	100	30	10	5	0.1	0.02	90	0.6

### Feature extraction and classification

For each leaf disk, areas of interest were delimited over asymptomatic zones and relevant BLSD spots using the MATLAB software (version 8.6.0, The MathWorks Inc., Natick, MA, 2015) and true colour visualisation of the hyperspectral images. For each of the 5941 pixel included in these areas of interest, the parameter values estimated from model inversion were extracted and associated with their disease stage. Note that *C*_*w*_ and *C*_*m*_ were not used here, as their influence on leaf reflectance in the VNIR range is very limited: the resulting *C*_*w*_ and *C*_*m*_ estimates thus provide little relevant information to discriminate the disease stages.

The *lda* multiclass Linear Discriminant Analysis (LDA) function from the MASS package^57^ of the R software (version 3.3.3, R Foundation for Statistical Computing, Vienna, Austria, 2017) was used to estimate the disease severity stage of our database’s pixels based on their inverted PROCOSINE parameter values. Linear discriminant analysis is a statistical technique that aims to describe, explain and predict membership of predefined groups (disease stage in our case) of a set of observations based on a series of predictive variables. It consists of maximizing the ratio of the between-class variance to the within-class variance using at most *n*−1 linear discriminant vectors, where *n* is the number of classes to be discriminated. Using these linear discriminant vectors, variables are projected into a sub-space where their assignment to a given class is determined based on the distance between the projected individual and the class centroids. Using the coefficients of the linear discriminant vectors, one can physically interpret the results of the LDA (in our case, the ecophysiological response of the banana leaf to the spread of the disease). Before performing the LDA, the explanatory variables (i.e., inverted parameters) were normalized using the following equation:

3 $$V{N}_{i}=\frac{{V}_{i}-mi{n}_{V}}{ma{x}_{V}-mi{n}_{V}}$$

where, for a given explanatory variable *V*, *VN*_*i*_ is the normalized value of *V* for the i^th^ pixel, *V*_*i*_ the value of *V* for the i^th^ pixel, and *min*_*V*_ and *max*_*V*_ are, respectively, the minimum and maximum values of *V* over all the 5941 pixels available in the database. Due to the structure and the size of our database, it was not possible to perform a random sampling to create the training and validation subsets, as pixels from the same disk would have been highly correlated. Consequently, the database was split into two independent subsets S1 (pixels belonging to the first disk of a given disease stage) and S2 (pixels belonging to the second disk of a given disease stage) of similar size (3294 pixels for S1 and 2647 for S2). We used a two-fold cross validation to compute the classification performances of the LDA for a variable number of discriminant axes, with a first model calibrated on S1 and validated on S2, and a second model calibrated on S2 and validated on S1.

The classification accuracy was assessed with producer accuracy, user accuracy and overall accuracy^58^. The producer accuracy (*PA*) of a given class is calculated as

4 $$PA=\frac{TP}{(TP+FN)}$$

where *TP* is the number of true positives for the considered class (each class is a disease stage), i.e., the number of pixels correctly classified into the considered class, and *FN* is the number of false negatives, i.e., the number of pixels belonging to the considered class but misclassified into another one. The user accuracy (*UA*) of a given class is given by

5 $$UA=\,\frac{TP}{(TP+FP)}$$

where *FP* is the number of false positives, i.e., the number of pixels incorrectly classified into the considered class. Finally, the overall classification accuracy is computed as follows:

6 $$accuracy=\frac{\sum TP}{{n}_{s}}$$

with *n*_*s*_ the number of samples in our dataset.

Finally, another LDA model was calibrated using the complete dataset to estimate more accurately the relative importance of each PROCOSINE parameter on the discriminant vectors.

## Electronic supplementary material

Supplementary material

## Data Availability

All data and codes used in this study are available upon request.
