# Supplementary material for: Exploring the potential of PROCOSINE and close-range hyperspectral imaging to study the effects of fungal diseases on leaf physiology
Source: Sci Rep. 2018 Oct 29;8:15933. doi: 10.1038/s41598-018-34429-0 (PMC6206143; doi:10.1038/s41598-018-34429-0)
Supplement: Supplementary file 1 — Supplementary material [file 41598_2018_34429_MOESM1_ESM.docx]

**Exploring the potential of PROCOSINE and close-range hyperspectral imaging to study the effects of fungal diseases on leaf physiology.**

**Julien Morel^1,2,*^, Sylvain Jay^3^, Jean-Baptiste Féret^4^, Adel Bakache^1^, Ryad Bendoula^1^, Francoise Carreel^5^, Nathalie Gorretta^1^.**

^1^UMR ITAP, Irstea, Montpellier SupAgro, Univ. Montpellier, Montpellier, France

^2^Department of Agricultural Research for Northern Sweden, Swedish University of Agricultural Sciences, Umeå, Sweden

^3^Aix-Marseille Univ., CNRS, Central Marseille, Institut Fresnel, Marseille F-13013, France

^4^UMR TETIS, Irstea, Univ. Montpellier, Montpellier, France

^5^UMR AGAP, Cirad, Montpellier, France

*julien.morel@irstea.fr


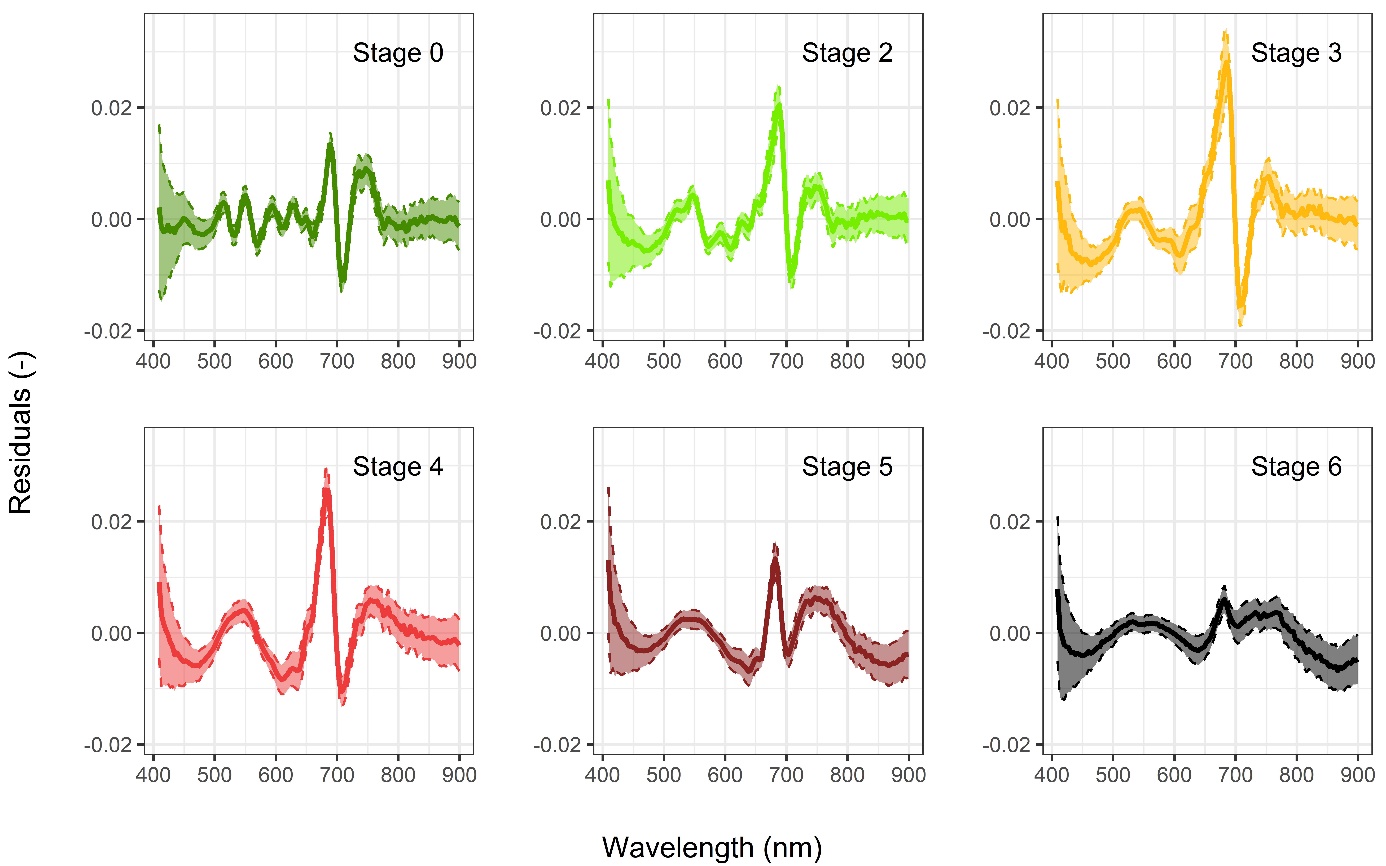


Figure 1. Mean (solid lines) and standard deviation (dashed lines) values of the error spectra (computed as the difference between the measured spectra and the simulated ones) for each disease stage according to the wavelength.


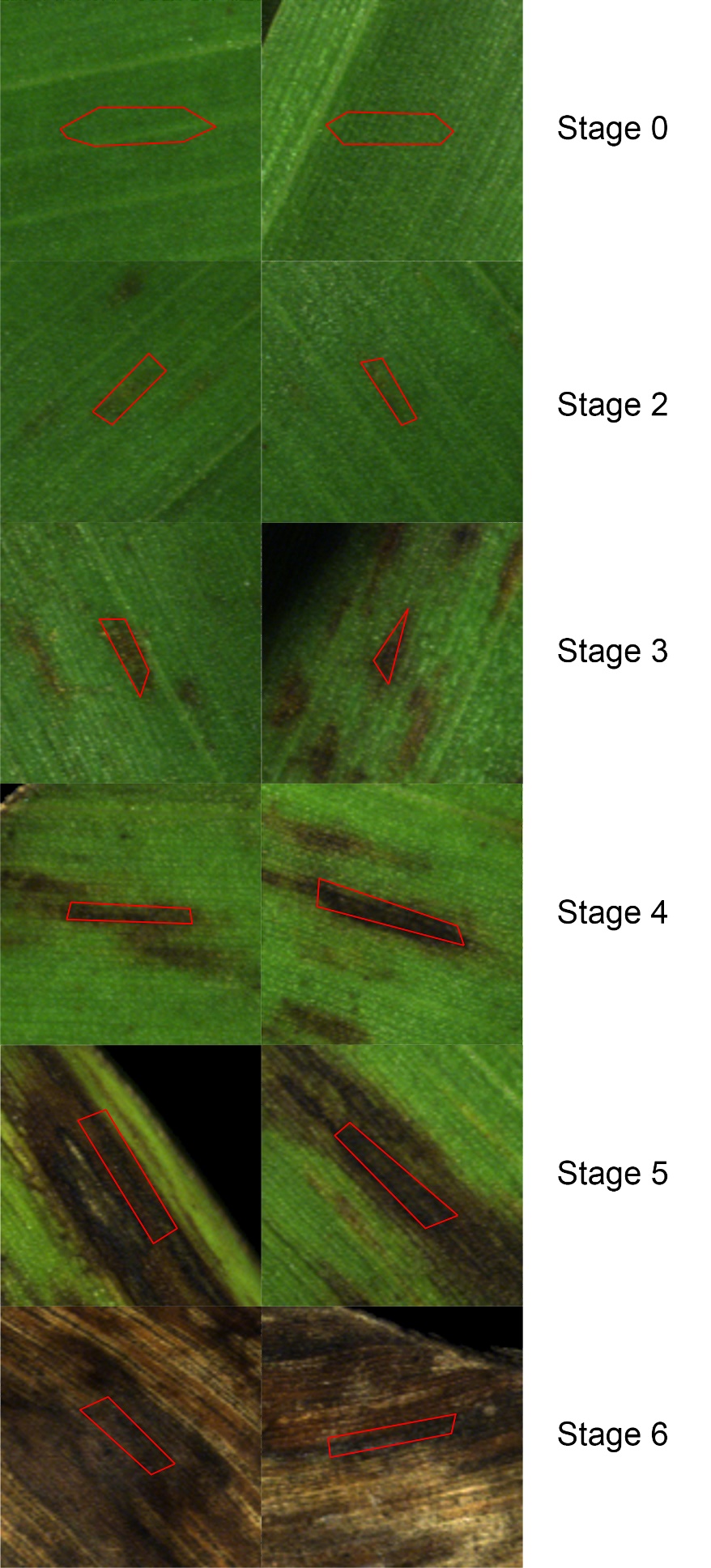


Figure 2. Areas of specific disease stages used in the study (red polygons).
